# Prediction of microvascular invasion in hepatocellular carcinoma based on preoperative Gd-EOB-DTPA-enhanced MRI: Comparison of predictive performance among 2D, 2D-expansion and 3D deep learning models

**DOI:** 10.3389/fonc.2023.987781

**Published:** 2023-02-03

**Authors:** Tao Wang, Zhen Li, Haiyang Yu, Chongfeng Duan, Weihua Feng, Lufan Chang, Jing Yu, Fang Liu, Juan Gao, Yichen Zang, Ziwei Luo, Hao Liu, Yu Zhang, Xiaoming Zhou

**Affiliations:** ^1^ Department of Radiology, Affiliated Hospital of Qingdao University, Qingdao, Shandong, China; ^2^ School of Medical Imaging, Weifang Medical University, Weifang, Shandong, China; ^3^ Yizhun Medical AI Co., Ltd, Beijing, China; ^4^ Department of Cardiology, Affiliated Hospital of Qingdao University, Qingdao, Shandong, China; ^5^ Department of Ultrasound, Affiliated Hospital of Qingdao University, Qingdao, Shandong, China

**Keywords:** microvascular invasion, hepatocellular carcinoma, gadoxetic acid-enhanced MRI, artificial intelligence, deep learning

## Abstract

**Purpose:**

To evaluate and compare the predictive performance of different deep learning models using gadolinium ethoxybenzyl diethylenetriamine pentaacetic acid (Gd-EOB-DTPA)-enhanced MRI in predicting microvascular invasion (MVI) in hepatocellular carcinoma.

**Methods:**

The data of 233 patients with pathologically confirmed hepatocellular carcinoma (HCC) treated at our hospital from June 2016 to June 2021 were retrospectively analyzed. Three deep learning models were constructed based on three different delineate methods of the region of interest (ROI) using the Darwin Scientific Research Platform (Beijing Yizhun Intelligent Technology Co., Ltd., China). Manual segmentation of ROI was performed on the T1-weighted axial Hepatobiliary phase images. According to the ratio of 7:3, the samples were divided into a training set (N=163) and a validation set (N=70). The receiver operating characteristic (ROC) curve was used to evaluate the predictive performance of three models, and their sensitivity, specificity and accuracy were assessed.

**Results:**

Among 233 HCC patients, 109 were pathologically MVI positive, including 91 men and 18 women, with an average age of 58.20 ± 10.17 years; 124 patients were MVI negative, including 93 men and 31 women, with an average age of 58.26 ± 10.20 years. Among three deep learning models, 2D-expansion-DL model and 3D-DL model showed relatively good performance, the AUC value were 0.70 (P=0.003) (95% CI 0.57–0.82) and 0.72 (P<0.001) (95% CI 0.60–0.84), respectively. In the 2D-expansion-DL model, the accuracy, sensitivity and specificity were 0.7143, 0.739 and 0.688. In the 3D-DL model, the accuracy, sensitivity and specificity were 0.6714, 0.800 and 0.575, respectively. Compared with the 3D-DL model (based on 3D-ResNet), the 2D-DL model is smaller in scale and runs faster. The frames per second (FPS) for the 2D-DL model is 244.7566, which is much larger than that of the 3D-DL model (73.3374).

**Conclusion:**

The deep learning model based on Gd-EOB-DTPA-enhanced MRI could preoperatively evaluate MVI in HCC. Considering that the predictive performance of 2D-expansion-DL model was almost the same as the 3D-DL model and the former was relatively easy to implement, we prefer the 2D-expansion-DL model in practical research.

## 1 Introduction

Hepatocellular carcinoma (HCC) is the most common primary malignant tumor of the liver (1). Surgery is currently considered the primary treatment for patients with hepatocellular carcinoma, yet postoperative recurrence and metastasis remain pressing challenges. There are many factors affecting the risk of recurrence of HCC after surgery, among which microvascular invasion (MVI) is a well-established independent risk factor for recurrence of HCC after surgical resection or liver transplantation (2–4). Microvascular invasion (MVI) is a nesting mass of cancer cells in the lumen of the vasculature lined with endothelial cells that can be observed by microscopy (5). It usually refers to affected vessels with a diameter of less than 300 μm, predominantly in small branches of the portal vein within the paracancerous tissue, and it is a marker of tumor aggressiveness. Several studies (6–8) have shown that in MVI-positive patients, hepatectomy with extended surgical margins can significantly improve patient survival by eradicating micrometastases. Since MVI can only be diagnosed by postoperative pathology, preoperative prediction of MVI is particularly important and will help clinicians select individualized treatment plans for HCC and thus reduce its early recurrence rate to a certain extent.

Many previous studies have predicted the occurrence of microvascular invasion of HCC based on the clinical characteristics and preoperative traditional imaging findings of HCC patients. These assessment indicators include relevant laboratory test results such as alpha fetoprotein (AFP) and total bilirubin (TBil). Imaging signs include tumor size, number, margin, capsule, peritumoral enhancement, peritumoral hypointensity in the hepatobiliary phase, etc. Some evaluation indicators are considered helpful for the preoperative prediction of MVI (9–11). However, the assessment of some traditional imaging signs often relies on the personal experience of radiologists and is inevitably subject to error, so conclusions from different studies are often inconsistent.

Recently, artificial intelligence (AI), which mainly consists of nondeep learning algorithms (NDLAs) and deep learning algorithms (DLAs), has been widely used in the medical field. Currently, radiomics based on nondeep learning algorithms is considered to be effective in predicting MVI by high-throughput extraction of a large number of quantitative imaging features for modeling (12). However, manual feature extraction is complex and time-consuming, and machine learning models constructed using different modeling approaches lack stability and consistent interpretation (13). In contrast to nondeep learning algorithms (NDLAs), deep learning algorithms (DLAs) are able to learn features directly from images instead of using artificially defined features based on human experience (14–16). Recently, Wang et al. (17) fused deep features extracted from multib-value DWI and ADC images to construct a deep learning model, which showed a better performance for MVI prediction. Although some researchers have begun to use deep learning algorithms to build models to predict the occurrence of liver cancer MVI, the dimensions of the models built by different researchers are different. To the best of our knowledge, there is no research comparing the difference in the predictive performance of MVI in HCC between 2D and 3D deep learning models based on gadoxetic acid disodium-enhanced MRI. Therefore, in this study, we constructed deep learning models with different dimensions to predict the occurrence of MVI in HCC to preliminarily explore the differences in predictive performance between different models.

## 2 Materials and methods

### 2.1 Study population

This retrospective study was approved by our institutional review board. Informed consent was waived. All identifying information of the included patients was deleted. Patients were identified by searching the electronic HIS (hospital information system) database at our hospital from June 2016 to June 2021. The demographic and pathologic data were collected from their electronic medical records. Finally, a total of 233 hepatocellular carcinoma patients were retrospectively selected for this study according to the following inclusion and exclusion criteria. The inclusion criteria were: (1) patients with histologically confirmed HCC after surgical resection; (2) patients with conclusive histopathological confirmation of their MVI status; and (3) preoperative Gd-EOB-DTPA-enhanced MRI performed within two weeks prior to surgery. The exclusion criteria were as follows: (1) patients with any previous antitumor treatment, including transarterial chemoembolization and radiofrequency ablation; (2) patients with unequivocal macrovascular invasion or metastasis; (3) MR images with poor quality (a low signal-to-noise ratio) that would affect the delineation of the region of interest (ROI).

### 2.2 MR image acquisition

A GE Signa HDx 3.0T MRI scanner with an 8-channel body phased-array coil was used to scan from the top of the diaphragm to the lower edge of the liver. The contrast agent for MRI contrast enhancement examination was gadolinium ethoxybenzyl diethylenetriamine pentaacetic acid (Gd-EOB-DTPA, Bayer Schering Pharma AG, Germany). A liver volume acceleration (liver acquisition with volume acceleration, LAVA) sequence 3D volume scan (TR 2.6 ms TE 1.2 ms) was applied, and the continuous acquisition of biarterial phase images began 15 s after the injection of the contrast agent, 45 s for portal phase images, 180 s for balancer phase images, and 20 min for hepatobiliary phase images. The dose of contrast agent was 0.1 mL/kg, and the injection flow rate was 1.0 mL/s with a rapid push through the elbow vein.

### 2.3 Development of DL models

**Figure 1:**
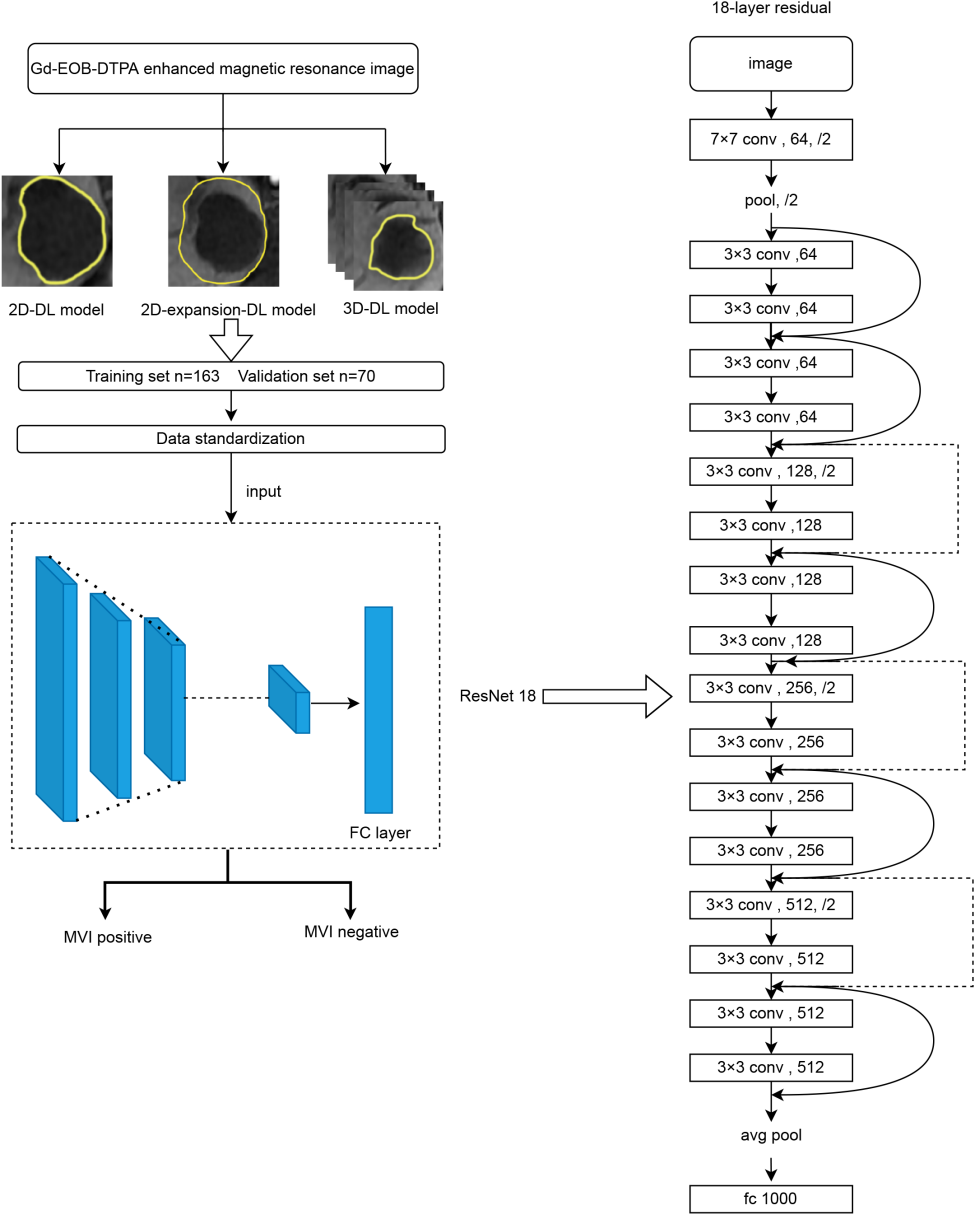
The architecture of the Deep Learning model.

The architecture of the DL model is shown in **Figure 1**. We adopted the ResNet18 convolutional neural network (CNN) as the primary branch for DL modeling. The hepatobiliary phase (HBP) images were selected as the input original image, and ROI was determined by manual segmentation on the hepatobiliary image. The images were assessed by radiologists with 5 years of experience in abdominal imaging under the supervision of a senior associate chief physician. The input ROIs were delineated using the Darwin Scientific Research Platform (Beijing Yizhun Intelligent Technology Co., Ltd., China) with three techniques: a three-dimensional delineation method in which the ROI was manually outlined on each axial slice of the hepatobiliary phase image covering the entire tumor, and a two-dimensional delineation method in which the tumor edge was completely outlined on the slice containing the largest diameter of the tumor. To further explore the intratumoral and peritumoral information, we used the standardized image morphological erosion and expansion method to expand the ROI obtained from the delineation method by 5 mm. This is what we call the two-dimensional expansion delineation method. Notably, segmentation should be discarded when the expanded area exceeds the liver or image edge. According to a ratio of 7:3, the samples were divided into a training set (N=163) and a validation set (N=70). The next step was to standardize the data. Data standardization means before the development of Deep Learning Models, the image was resampled (each voxel to 1×1×1 mm^3^) and the gray value was normalized using the Darwin Scientific Research Platform (Beijing Yizhun Intelligent Technology Co., Ltd., China). Finally, the training set was used to train the deep learning model and evaluate the prediction performance of the model (using the validation set).

### 2.4 Statistical analysis

SPSS 22.0 (Chicago, IL, USA) software was used for statistical analysis. Categorical variables are represented as numbers or percentages. Continuous variables are expressed as the mean ± standard deviation, and comparisons among the categorical data were performed by chi-square tests. For numerical variables that conform to the normal distribution, independent student t-tests were used, if not, Mann-Whitney tests were used.

Parameters such as the total floating point operations (total flops) and frames per second (FPS) among different deep learning models are obtained from the Darwin Scientific Research Platform (Beijing Yizhun Intelligent Technology Co., Ltd., China). Receiver operating characteristic curve (ROC) analysis was used to evaluate the predictive performance of the three models, calculating the area under the curve (AUC), sensitivity, specificity, and accuracy. The difference in predictive performance between different models was compared using the Delong test. Bilateral tests were used for all statistical tests, and P < 0.05 was considered statistically significant.

## 3 Results

Among 233 HCC patients, 109 were pathologically MVI-positive, including 91 men and 18 women, with an average age of 58.20 ± 10.17 years; 124 patients were MVI-negative, including 93 men and 31 women, with an average age of 58.26 ± 10.20 years. **Table 1** summarizes the demographic features compared between the MVI-positive and MVI-negative groups. Compared with patients without MVI, patients with MVI had larger tumor sizes.

**Table 1:** The demographic features between MVI positive and MVI negative groups.

	MVI-Negative (n=124)	MVI-Positive(n=109)	P
Sex			0.113
Female	31	18	
Male	93	91	
Age, years	58.26 ± 10.20	58.20 ± 10.17	0.902
Maximum tumor diameter (mm)	38.02 ± 22.58	56.84 ± 33.17	<0.001

**Figure 2:**
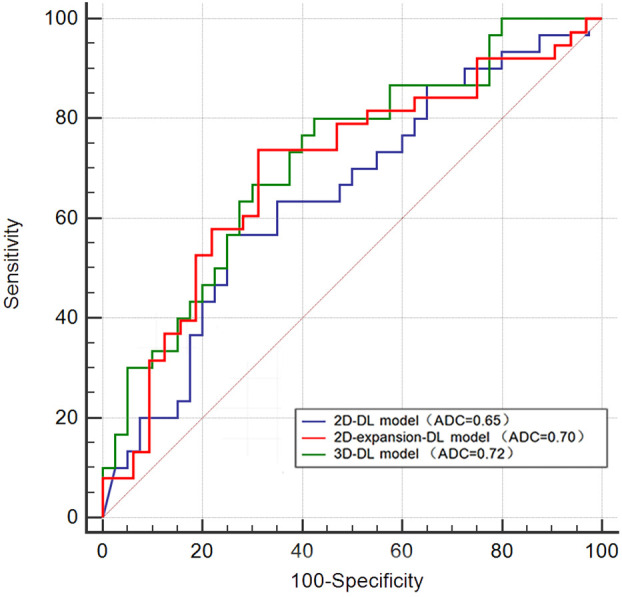
The ROC curves of the three models in the testing set.

In the 2D-DL model, the AUC for predicting MVI was 0.81 (95% confidence interval (CI) 0.74–0.87) in the training set and 0.65 (95% CI 0.52–0.78) in the testing set. In the training set, the accuracy, sensitivity and specificity were 0.7301, 0.785 and 0.679, respectively. In the testing set, the accuracy, sensitivity and specificity were 0.6714, 0.567 and 0.750, respectively. In the 2D-expansion-DL model, the AUC for predicting MVI was 0.82 (95% confidence interval (CI) 0.76–0.89) in the training set and 0.70 (95% CI 0.57–0.82) in the testing set. In the training set, the accuracy, sensitivity and specificity were 0.7716, 0.835 and 0.690, respectively. In the testing set, the accuracy, sensitivity and specificity were 0.7143, 0.739 and 0.688, respectively. In the 3D-DL model, the AUC for predicting MVI was 0.77 (95% confidence interval (CI) 0.70–0.84) in the training set and 0.72 (95% CI 0.60–0.84) in the testing set. In the training set, the accuracy, sensitivity and specificity were 0.7362, 0.881 and 0.582, respectively. In the testing set, the accuracy, sensitivity and specificity were 0.6714, 0.800 and 0.575, respectively (**Table 2**). The Delong test showed that the AUCs of the 2D-DL model and 2D-expansion-DL model were not significantly different, with a P value of 0.681 (>0.05). Similarly, the AUCs of the 2D-DL model and 3D-DL model were not significantly different, with a P value of 0.405 (>0.05) (**Figure 2**).

**Table 2:** Performance of different DL models in the training and the testing set.

Model		AUC (95% CI)	Sensitivity (95% CI)	Specificity (95% CI)	Accuracy
2D-DL model	TR	0.81 (0.74-0.87)	0.785 (0.682-0.861)	0.679 (0.573-0.769)	0.7301
	TE	0.65 (0.52-0.78)	0.567 (0.392-0.726)	0.750 (0.598-0.858)	0.6714
2D-expansion-DL model	TR	0.82 (0.76-0.89)	0.835 (0.746-0.897)	0.690 (0.575-0.785)	0.7716
	TE	0.70 (0.57-0.82)	0.739 (0.580-0.850)	0.688 (0.514-0.820)	0.7143
3D-DL model	TR	0.77 (0.70-0.84)	0.881 (0.795-0.934)	0.582 (0.472-0.685)	0.7362
	TE	0.72 (0.60-0.84)	0.800 (0.627-0.905)	0.575 (0.422-0.715)	0.6714

AUC, area under the curve; CI, confidence interval; DL, deep learning; TR, training set; TE, testing set.

The total floating point operations (total flops) and frames per second (FPS) among the different deep learning models are shown in **Table 3**. FPS is a common metric used to evaluate the speed of a model, and it indicates the number of images that the model can process per second. The FPS for the 2D-DL model (based on 2D-ResNet) is 244.7566, which is much larger than that of the 3D-DL model (73.3374).

**Table 3:** The Total Flops and FPS between the 2D and 3D models.

	2D-model (based on 2D-Resnet)	3D-model (based on 3D-Resnet)
Total Flops	1.82GFlops	10.73GFlops
FPS	244.7566	73.3374

## 4 Discussion

The preoperative assessment of microvascular invasion of liver cancer has always been a challenging area in the field of medical imaging. Myata et al. (18) reported that patients who suffered from hepatocellular carcinoma with microvascular invasion had a more than 4-fold increased risk of tumor recurrence. Compared with ordinary gadolinium contrast agents, Gd-EOB-DTPA can not only display the blood supply of the lesion but also reveal the hepatocyte function (19). In the hepatobiliary phase of the MRI enhancement scan, the surrounding liver parenchyma shows an increased signal because of the uptake of contrast agents. The expression of organic anion transporting polypeptide 8 (OATP-8) in most HCCs gradually decreases during the process of hepatocarcinogenesis; thus, the tumor cells do not uptake contrast and show a low signal on Gd-EOB-DTPA enhancement images. Currently, Gd-EOB-DTPA-enhanced MRI has been applied to predict microvascular invasion in hepatocellular carcinoma by some researchers (20), in addition to its routine use in the imaging diagnosis of hepatocellular carcinoma.

With the establishment of large medical databases and the development of computer hardware, artificial intelligence technology represented by deep learning has entered the field of medical image diagnosis. By recognizing and classifying medical images, it can discover imaging features that cannot be identified or are ignored by radiologists, especially deep learning models based on convolutional neural networks (CNNs), which have shown excellent performance in medical image recognition (21). Many researchers have predicted the occurrence of MVI in hepatocellular carcinoma preoperatively by using deep learning models. As one of the representative cases, Zhang et al. (22) established and verified four 3D CNN-based deep learning models based on MRI images to predict microvascular invasion in HCC before surgery. A fusion model combining T2WI, T2-SPIR and PVP images achieved better performance than a single image-based model in predicting the MVI status of HCC patients. Wei J et al. (20) developed deep learning models based on enhanced CT (CE-CT) and EOB-MRI for the preoperative assessment of MVI and prospectively validated the effectiveness of two deep learning models. The results showed that the EOB-MRI-based deep learning model was better than the enhanced CT-based deep learning model in predictive performance. However, different groups have chosen different classification networks to build their models, and the dimensions of the models that have been constructed are also different. The ResNet network, as the mainstream deep learning network, has a directly connected channel in the residual structure that can skip one or several layers, and the information in the shallow layer can be directly input to the deeper layers. The network only needs to learn the residuals of the previous network output, thus effectively avoiding the problem of gradient explosion and allowing the network to be trained at a deep level. In addition, Han et al. (23) also pointed out that deep learning CNN models, such as ResNet, when pretrained on the ImageNet dataset can be beneficial for the visual recognition task of medical images. Based on the above, the author used the ResNet18 classification network to construct 2D, 2D-expansion, and 3D deep learning models to predict MVI in hepatocellular carcinoma and determined whether there was a difference in predictive performance among the different models.

For the 2D deep learning model, the AUC for predicting MVI was 0.65 (95% CI 0.52–0.78) in the testing set, slightly inferior to the 3D deep learning model (0.72 (95% CI 0.6–0.84)). However, the Delong test showed that the AUC of the two models was not significantly different, with a P value of 0.405 (>0.05), meaning that the predictive efficacy of the two models for microvascular invasion in hepatocellular carcinoma was not significantly different. Why does the 3D-DL model, which theoretically carries more information about liver cancer lesions, not show a significant advantage in predicting MVI compared with the information-impaired 2D-DL model? In the author’s opinion, the main purpose of acquiring 3D data to construct a deep learning model is to capture the gradient information between the layers formed by a lesion spanning multiple consecutive layers to effectively combine the overall information from consecutive layers of the lesion. However, for our dataset, the layer thicknesses along the z-axis are large (5 mm), and only limited contextual information can be obtained from the z-axis. For such data, the largest information discrepancy tends to occur in one plane, the axial plane. At this time, the advantage of the 3D-DL model, which theoretically carries more information about the lesion, is not obvious compared with the 2D-DL model. To further explore the intratumoral and peritumoral information, we used the standardized image morphological erosion and expansion method to delineate the ROI and obtain the 2D-expansion deep learning model. The AUC and sensitivity of the 2D-expansion-DL model were higher than those of the 2D-DL model and were almost the same as those of the 3D-DL model.

In addition, comparing the difference in performance between different deep learning models, we have to mention the running speed of the models. FPS is a common metric used to evaluate the speed of a model, and it indicates the number of images that the model can process per second. Therefore, a larger FPS indicates faster processing and requires less computation and execution time for the model. In our study, the FPS for the model based on the 2D-ResNet18 classification network was 244.7566, which is much larger than that of the 3D-ResNet18 model (73.3374). Liu et al. (24) showed that the 2D-DL model based on ResNet18 ran faster on the GPU than the 3D model, and the memory occupied by the 2D-DL model was 50% less than that of the 3D model. That is, the efficiency of the 2D-DL model is higher than that of the 3D-DL model. Our findings are consistent with the above. Moreover, relevant literature (25, 26) indicates that a 3D-DL model is larger than the 2D-DL model, more parameters need to be adjusted when building a 3D deep learning model, and running a 3D deep learning model is more time-consuming and requires more training data and storage space. From this point of view, on the premise of meeting the requirements of the task, the 2D-DL model, which is small in scale and faster in operation, has lower requirements for hardware and higher model applicability.

In conclusion, the deep learning model based on gadoxetate disodium-enhanced MRI has a certain value in predicting the microvascular invasion of hepatocellular carcinoma. Based on the ResNet18 classification network, the AUC and sensitivity of the 2D-expansion-DL model were almost the same as those of the 3D-DL model. Considering that building a 2D-expansion-DL model is relatively easy to implement while ensuring its predictive performance, we prefer the 2D-expansion-DL model in practical research.

Nevertheless, the present study also has some limitations. (1) This study is a single-center study, and internal validation was used to evaluate the predictive performance of the model. A multicenter prospective study will be conducted later, using external validation to improve the predictive performance of the model. (2) Only the value of Gd-EOB-MRI images in predicting microvascular invasion in hepatocellular carcinoma was investigated, and the predictive value of clinical features and qualitative imaging features were not considered. Because this was not the focus of this study, further integration of clinical and conventional imaging features will be used to construct a model in future studies.

## Data availability statement

The raw data supporting the conclusions of this article will be made available by the authors, without undue reservation.

## Author contributions

TW, ZL, WF and XZ designed the study. HY, CD, LC, JY, FL, JG, YZ and HL performed the statistical analysis. CD, YZ and ZL drafted the figure. TW and ZL drafted the manuscript. WF and XZ supervised the study. All authors contributed to the article and approved the submitted version.
